# Characterization of the peripheral blood transcriptome and adaptive evolution of the MHC I and TLR gene families in the wolf (*Canis lupus*)

**DOI:** 10.1186/s12864-017-3983-0

**Published:** 2017-08-07

**Authors:** Guangshuai Liu, Honghai Zhang, Guolei Sun, Chao Zhao, Shuai Shang, Xiaodong Gao, Tian Xia, Xiufeng Yang

**Affiliations:** Qufu Normal University, Jingxuan Street No. 57, Qufu, Shandong province China

**Keywords:** Wolf, Blood transcriptome, RNA-seq, Immune system, MHC, TLR

## Abstract

**Background:**

The wolf (*Canis lupus*) is one of the most widely distributed terrestrial mammals, because it is well adapted to various ecological niches and their corresponding pathogen environments. Immunological competence is a crucial factor involved in adapting to a changing environment and fighting pathogen infection in animals. In this study, the peripheral blood transcriptome of wolves was generated via RNA-seq to advance understanding of the wolf immunome, with a special focus on the major histocompatibility complex class I (MHC I) and toll-like receptor (TLR) gene families, which are involved in pathogen recognition and defense.

**Results:**

The blood transcriptomic libraries of eight wolves originating from Tibet and Inner Mongolia were sequenced, and approximately 383 million reads were generated. Using a genome-guided assembly strategy, we obtained 123,851 unigenes, with a mean length of 845 bp and an N50 length of 1121 bp. On the basis of BLAST searches against the NCBI non-redundant protein database (Nr), a total of 36,192 (29.22%) unigenes were annotated. For functional classification, 24,663 unigenes were assigned to 13,016 Gene Ontology (GO) terms belonging to 51 sub-categories of the three main GO categories. Additionally, 7682 unigenes were classified into 6 Kyoto Encyclopedia of Genes and Genomes (KEGG) categories, in which the most represented functional sub-categories were signal transduction and the immune system, and 16,238 unigenes were functionally classified into 25 Eukaryotic Orthologous Groups (KOG) categories. We observed an overall higher ω (*d*_N_/*d*_S_) value at antigen-binding sites (ABSs) than at non-ABS regions as well as clear evidence of intergenic/intragenic recombination events at wolf MHC I loci. Additionally, our analysis revealed that carnivorous TLRs were dominated by purifying selection, with mean ω values at each TLR locus ranging from 0.173 to 0.527. However, we also found significant instances of positive selection that acted on several codons in pathogen recognition domains and were linked to species-specific differences in pathogen recognition.

**Conclusions:**

This study represents the first attempt to characterize the blood transcriptome of the wolf and to highlight the value of investigating the immune system. Balancing selection and recombination have contributed to the historical evolution of wolf MHC I genes. Moreover, TLRs in carnivores have undergone adaptive evolution against the background of purifying selection, and a high level of adaptive evolution was detected in the wolf TLR system.

**Electronic supplementary material:**

The online version of this article (doi:10.1186/s12864-017-3983-0) contains supplementary material, which is available to authorized users.

## Background

The gray wolf (*Canis lupus*) is one of the world’s most widely distributed mammals and is found in a wide range of ecologically different habitats [1]. This distribution suggests that the wolf is exposed to a complex pathogenic environment, which might drive the evolution of immune-related genes. Immunological competence is crucial for animals to adapt to a changing environment and to fight pathogen infection. Hence, illustrating the nature of the immune system is essential to explore the immunological responses and disease resistance ability of wolves, and effective conservation efforts often make use of immunological insights [2, 3]. Furthermore, dogs are an increasingly important model system for understanding the genetic basis of phenotypic diversity [4] and the pathogenesis of many human diseases [5, 6]. Given the unusual evolutionary relationships between wolves and dogs, documenting the immune architecture of wolves can also provide insights into the adaptive changes that occurred in dogs during the domestication process.

Blood acts as the pipeline of the immune system, carrying various immunomodulatory factors and specific antibodies that are involved in innate immune defenses as well as in the initiation of adaptive immune responses [7]. Fluctuations in the expression profiles of genes in the blood recapitulate information about genetic, epigenetic, cellular and environmental factors [8]. Therefore, blood constitutes a particularly attractive source of surrogate tissue for assessing the status of the immune system, which best monitored by profiling transcript abundance in blood. Indeed, blood transcriptome profiling has been developed into a mainstream tool for exploring the pathogenesis of a wide range of human diseases [8, 9]. Many molecular and cellular profiling approaches are currently available for the trancriptome profiling analyses. As compared with other technologies, sequence-based transcriptomic assays, such as RNA-seq, enable genome-wide analysis of the complexity of whole transcriptional landscapes with the greatest breadth and robustness [10]. Blood transcriptome profiling has been successfully performed on several wild mammal species, such as koala [11], giant panda [12], polar bear and brown bear [13]. However, no studies addressing the wolf blood transcriptome have been published to date.

Pathogens are among the most remarkable selective forces acting on wildlife during their evolutionary history. Detailed investigation of evolutionary patterns in genes involved in pathogen recognition is crucial for understanding coevolutionary processes between pathogens and their hosts [14]. Thus, two pathogen recognition receptor gene families, the major histocompatibility complex (MHC) and the toll-like receptors (TLRs), which play central roles in the adaptive and innate immune systems of vertebrates, respectively, were selected for analysis in the present study. The MHC loci constitute a highly polymorphic region of the mammal genome, and variants of these genes have been associated with pathogen resistance and the long-term survival of species [15]. Three major groups of MHC genes have been distinguished in mammals [16]. The MHC class I and II genes encode cell-surface glycoproteins that present pathogen-derived antigens to effector cells and are involved in triggering the downstream adaptive immune cascade [15, 17]. Balancing selection is commonly accepted to be responsible for the maintenance of polymorphism at these loci in a wide range of taxa [18]. Recombination is also presumed to be an important evolutionary mechanism driving MHC allelic diversity and polymorphism [19]. A considerable amount of research has previously been conducted on wolf MHC II loci [20–22]. However, no assessment of the adaptive pattern of wolf MHC I loci has been performed to date. Seven MHC class I genes have been identified in the dog genome, among which four are transcribed and are designated functional genes: DLA-12, DLA-64, DLA-79 and DLA-88 [23, 24].

TLRs encode type I transmembrane glycoproteins that recognize a wide diversity of pathogens by sensing pathogen-associated molecular patterns (PAMPs), and they can be classified as either non-viral (TLR1, TLR2, TLR4, TLR5, TLR6 and TLR10) or viral TLRs (TLR3, TLR7, TLR8 and TLR9) according to ligand specificity in many mammals [25]. The general structure of TLRs is characterized by a variable number of pathogen-recognition extracellular leucine-rich repeats (LRRs), a transmembrane region and a conserved intracellular toll/interleukin-1 receptor (TIR) signaling domain [26]. Because TLRs lie directly at the host-pathogen interface, they are potentially subject to coevolutionary dynamics. TLRs are evolutionarily conserved across vertebrates. However, some studies on primates [27], rodents [28] and birds [29] have revealed different degrees of species-specific positive selection acting on TLR repertory. At present, the role of pathogen-mediated selection in the evolution of TLRs among carnivores remains unknown.

To obtain a comprehensive understanding of the immunological properties of wolves, we first performed genome-wide transcriptome sequencing of peripheral blood by using RNA-seq. Then, a powerful genome-guided de novo assembly strategy was implemented in Trinity software [30] and used to generate a high-quality transcriptome. We utilized the obtained sequence dataset to perform a general characterization of the blood transcriptome with regard to gene functional annotation, immune pathway identification and the expression levels of immune-related genes. Furthermore, we studied the evolutionary patterns of two immune receptor gene families (MHC I and TLRs) and attempted to reveal the adaptive mechanism of the wolf response to a complex pathogen environment. To the best of our knowledge, this study is the first attempt to characterize the peripheral blood transcriptome of the wolf.

## Results

### Illumina sequencing and assembly

A total of 383 million paired reads with a length of 125 bp were generated from eight blood transcriptome libraries. After trimming and quality filtering, 373 million (97.32%) high-quality clean reads were retained and were aligned to the dog reference genome (CanFam3.1). In total, 146,640 transcripts were collected and assembled into 123,851 unigenes with an average length of 845 bp and an N50 length of 1121 bp (Table 1). All of the clean reads were then aligned back to the unigenes to assess the read content of the assembly. The results showed that up to 83.7% of the reads mapped back to the unigenes, thus indicating that the assembled unigenes represented the transcriptome reads well.

**Table 1:** Summary of the wolf blood transcriptome sequencing and assembly

Category	Numbers
Total raw reads	383,315,270
Total clean reads	373,038,916
Total bases (bp)	46,629,864,500
Total assembled transcripts	146,640
Total assembled unigenes	123,851
Total length of unigenes (bp)	104,598,095
Max length of unigenes (bp)	18,148
Min length of unigenes (bp)	301
Average length of unigenes (bp)	845
Contig N10 (bp)	4836
Contig N50 (bp)	1121

### Annotation of unigenes

All of the unigenes were annotated by using BLAST to Nr, NCBI non-redundant nucleotide database (Nt), KOG, a manually annotated and reviewed protein sequence database (Swiss-Prot), KEGG, and GO databases. Results showed that the Nt database has the largest matches with 81,101 (65.5%) unigenes were annotated. In total, 44,576 (36.0%) unigenes had matches in the five public protein databases, and the 36,192 (29.22%) unigenes BLAST hits in the Nr database were studied as the Nr database had the maximum protein reference sequences (Additional file 1: Fig. S1A). The species distribution of the best BLAST hits was shown in Additional file 1: Fig. S1B, and expectedly, most annotated unigenes had hits to the canine (22,573, 62.4%). The E-value distribution of matches showed that 13,271 (36.7%) unigenes have a highly significant homology (≤ 1.0E-100) and 3409 (9.4%) unigenes have E-value greater than 1E-10 (Additional file 1: Fig. S1C). The identity distribution of unigenes revealed that 28,208 (77.9%) unigenes have a high similarity (>80%) and 32,886 (90.9%) unigenes have a similarity greater than 60% (Additional file 1: Fig. S1D).

### Functional annotation and classification results

**Fig. 1:**
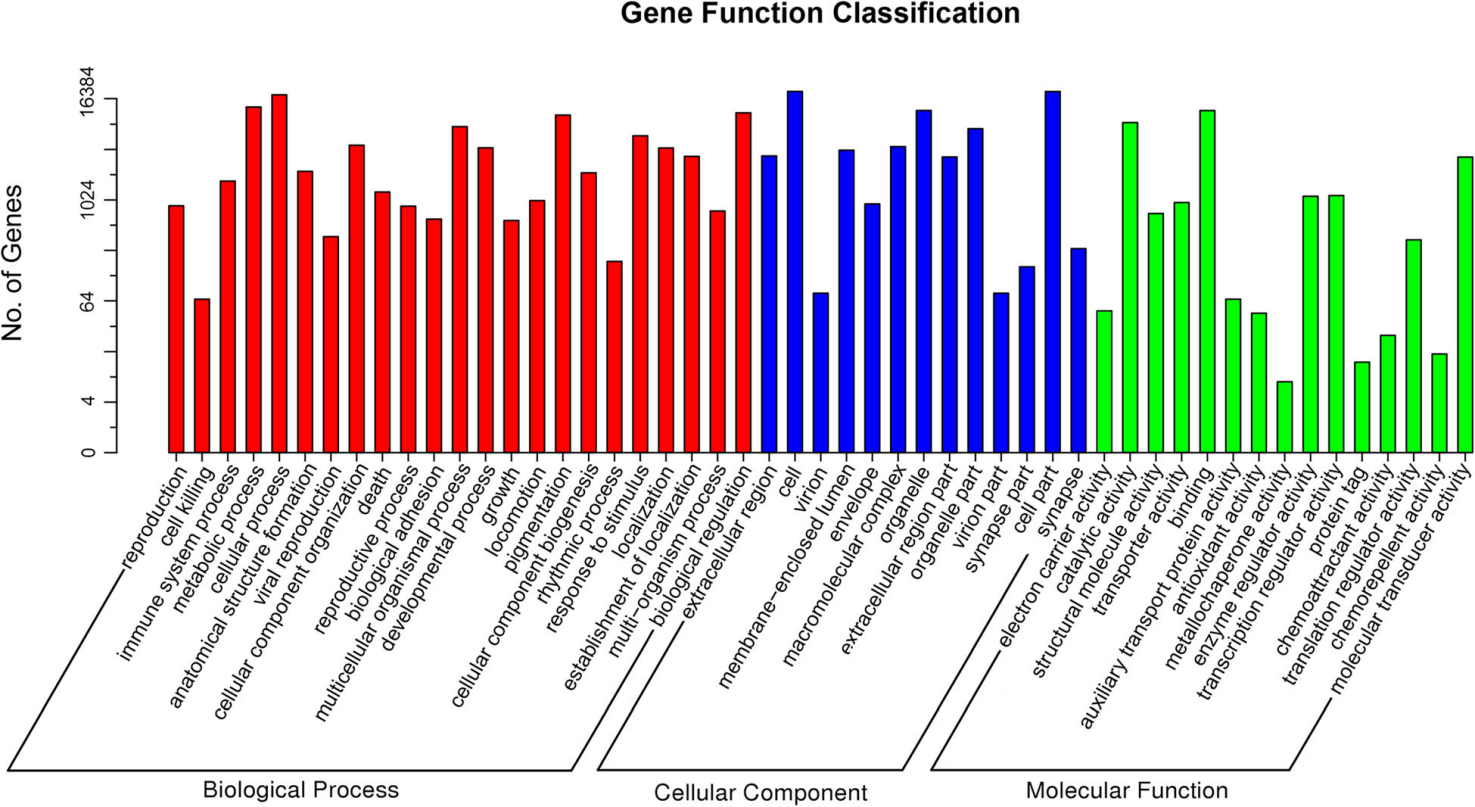
Histogram of the GO classification of unigenes

On the basis of sequence homology against the Nr database, a total of 24,663 unigenes were annotated to 13,016 GO terms, belonging to 51 GO sub-categories (Fig. 1). There were 23 sub-categories in BP (Biological Process), 13 sub-categories in CC (Cellular Component), and 15 sub-categories in MF (Molecular Function), respectively. Cellular process (GO: 0009987) and the metabolic process (GO: 0008152) were the most represented BP categories (Additional file 2: Table S1).

**Fig. 2:**
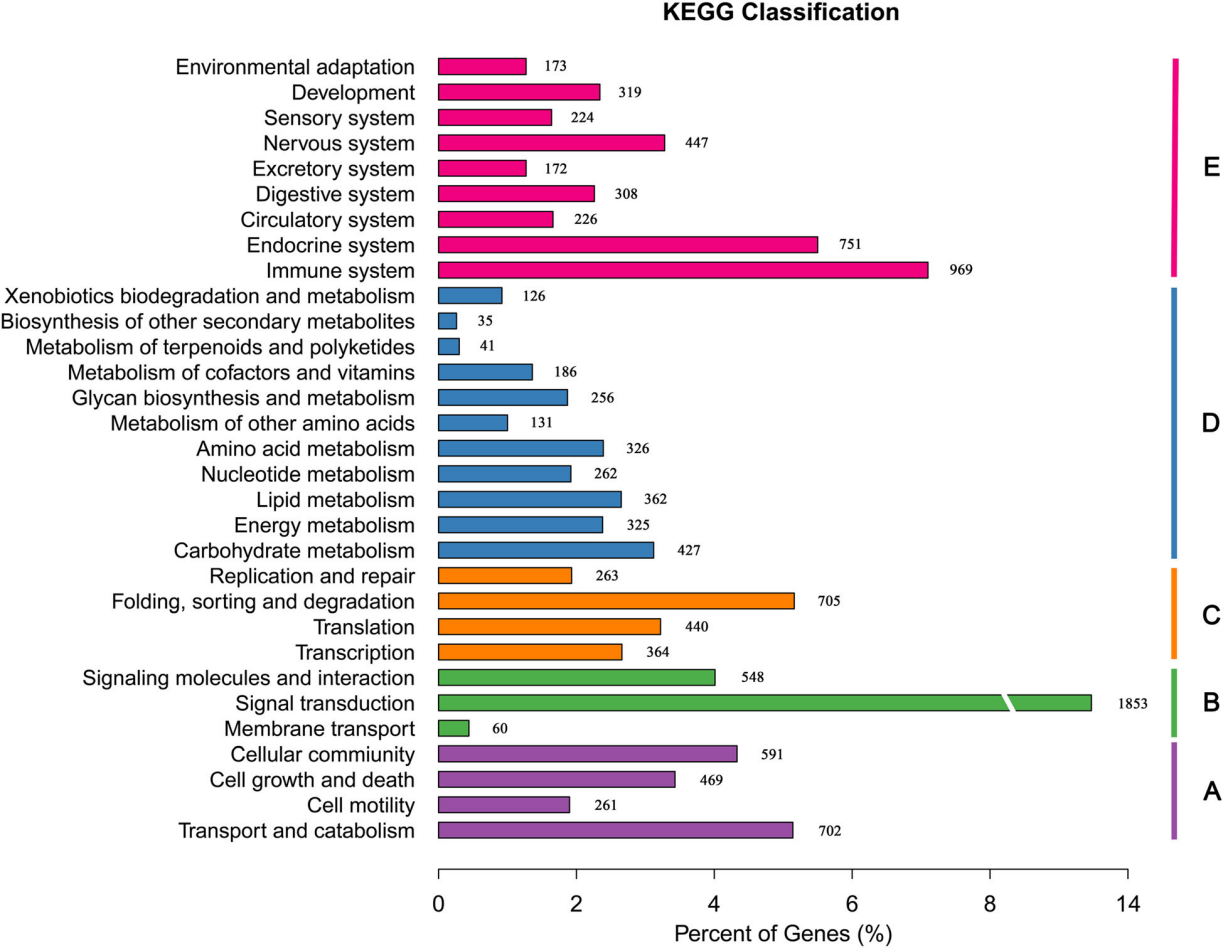
Histogram of the KEGG functional classification of unigenes. A: Cellular Processes, B: Environmental Information Processing, C: Genetic Information Processing, D: Metabolism, E: Organismal Systems

KEGG analysis showed that a total of 13,665 unigenes had significant matches in the database, and 7682 unigenes were functionally classified into 6 KEGG categories, including 335 KEGG Orthology (KO) pathways (Additional file 3: Table S2). Among the 31 identified sub-categories within the KEGG categories excluding human diseases, signal transduction (1853) and the immune system (969) were the most highly represented categories (Fig. 2). Within the organismal systems functional category, it was notable that the immune system represented 969 of the 2230 unigenes involved in 15 immune-related KEGG pathways (Table 2, Additional file 4: Table S3). With the exception of the platelet activation pathway, the annotated immune-related pathways contained 79.0% mapped genes among the total known genes in the pathways. Among the identified immune pathways, the chemokine signaling pathway (ko04062) was the most represented. Pathways involving the MHC and TLR gene families were also identified, which included antigen processing and presentation (ko04612), the intestinal immune network for IgA production (ko04672) and natural killer cell-mediated cytotoxicity (ko04650) for MHC and the toll-like receptor signaling pathway (ko04620) for TLRs. Pathways acting as a functional bridge between the innate and adaptive immune responses were also identified, such as Fc gamma R-mediated phagocytosis, NOD-like receptor and RIG-I-like receptor signaling pathways [31–33] (Table 2, Additional file 4: Table S3).

**Table 2:** Immune-related pathways identified in the wolf blood transcriptome

KO identifier	Pathway name	Annotated unigenes	Mapped genes	Genes in pathway
ko04062	Chemokine signaling pathway	216	114	146
ko04660	T cell receptor signaling pathway	166	77	85
ko04670	Leukocyte transendothelial migration	155	60	75
ko04620	Toll-like receptor signaling pathway	136	67	76
ko04650	Natural killer cell mediated cytotoxicity	132	66	81
ko04666	Fc gamma R-mediated phagocytosis	121	50	58
ko04640	Hematopoietic cell lineage	98	63	77
ko04621	NOD-like receptor signaling pathway	93	39	51
ko04662	B cell receptor signaling pathway	92	52	57
ko04612	Antigen processing and presentation	91	36	41
ko04664	Fc epsilon RI signaling pathway	87	39	45
ko04622	RIG-I-like receptor signaling pathway	79	46	53
ko04610	Complement and coagulation cascades	75	50	78
ko04623	Cytosolic DNA-sensing pathway	62	41	51
ko04672	Intestinal immune network for IgA production	59	28	37

**Fig. 3:**
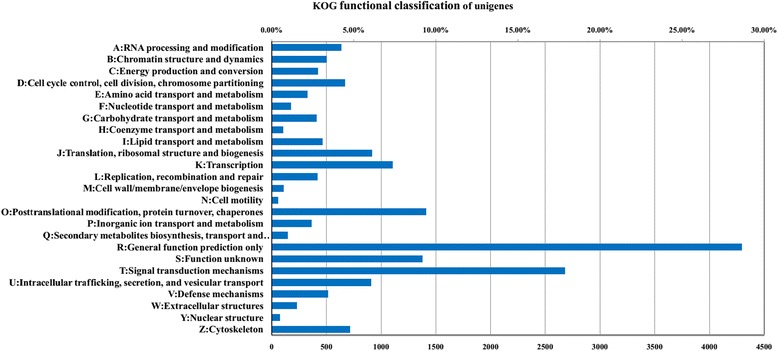
Histogram of the KOG functional classification of unigenes

By KOG classifications, 16,238 unigenes were annotated into 25 categories (Fig. 3). The cluster of general function prediction only exhibited 4296 (26.46%) unigenes, which constituted the most represented functional group. Signal transduction mechanism (2676, 16.48%) was in the second place, followed by posttranslational modification, protein turnover, chaperones at 1409 (8.68%) and function unknown at 1376 (8.47%). (Additional file 5: Table S4).

### Evidence of positive selection and recombination in the MHC I gene family

The complete sequences encoding the antigen-binding domains of 4 MHC I genes were amplified from the cDNA of 4 blood samples that were also used in the transcriptome analyses. A total of 17 alleles (exon 2-exon 3) were identified among the 4 MHC I loci, and the number of alleles at each locus was a follows: 6 (DLA-12), 2 (DLA-64), 2 (DLA-79) and 7 (DLA-88) [GenBank: KX583612-KX583628; Additional file 6: Fig. S2]. Among these alleles, 2 DLA-12, 1 DLA-64, 1 DLA-79 and 6 DLA-88 alleles have not been identified previously in any canid species. On the basis of the BLAST results in terms of Nr and FPKM (fragments per kilobase of transcript per million fragments sequenced) values, we identified 4 and 12 transcripts of MHC I and MHC II genes, respectively, in the wolf blood transcriptome. Among the identified MHC genes, DLA-64 and DLA-DRB1 were the most highly expressed MHC I and MHC II genes, with FPKM values of 371 and 291, respectively (Additional file 7: Table S5).

Evidence of historical positive selection was detected in the ABSs (encoded by exon 2 and exon 3) of DLA-12 and DLA-88 (Table 3), corresponding to a pattern expected for MHC genes under balancing selection. Regarding the putative ABS of DLA-88, the rate of nonsynonymous substitutions (*d*_N_) was significantly higher than the rate of synonymous substitutions (*d*_S_) by more than 3-fold (ω = 3.3; *p* = 0.002). A ω value greater than 1 was also detected at non-ABS positions, but this difference showed no significant deviation from neutrality (Table 3). Moreover, *d*_N_ was 13.6 times higher in ABSs than in non-ABSs. Surprisingly, the ω value of the ABSs of DLA-12 was not significant (*p* = 0.313) contrary to the non-ABSs, although *d*_N_ was higher than *d*_S_ in both the ABSs and the non-ABSs, probably because of a high level of nonsynonymous activity in non-ABSs or a conservative ABS position classification method. Notably, the variants in DLA-12 alleles were all nonsynonymous changes (Additional file 6: Fig. S2). CODEML analysis showed that the M8 model fit the data better than the M7 model (Table 3). In a comparison of M8 and M7 site models, we detected 15 positively selected sites from MHC I genes, among which 10 sites were located at ABSs. Moreover, 8 and 11 codons were inferred to be positively selected sites in DLA-12 and DLA-88, respectively, among which 1 and 7 codons were located in ABSs (Table 3, Additional file 6: Fig. S2).

**Table 3:** Rates of *d*_N_ and *d*_S_ substitutions in the ABSs and non-ABSs of wolf MHC I genes

		N	*d*_N_	*d*_S_	Z	*p*	-2lnΔL	PAML M8
MHC I	ALL sites	181 or 182	14.5 (1.5)	10.2 (1.4)	**2.304**	**0.023**	104.28**	44, 61, 65, 66, 72, 73, 95, 97, 99, 114, 116, 138, 152, 156, 157
	ABS	29	41.6 (8.7)	20.0 (6.2)	**2.69**	**0.008**		
	Non-ABS	152 or 153	10.6 (1.3)	8.6 (1.4)	1.063	0.29		
DLA-12	All sites	181	0.9 (0.3)	0	**2.834**	**0.005**	14.15**	49, 51, 61, 114, 116, 127, 141, 154
	ABS	29	0.6 (0.5)	0	1.013	0.313		
	Non-ABS	152	0.9 (0.3)	0	**2.66**	**0.009**		
DLA-88	All sites	182	4.8 (1.0)	2.1 (0.6)	**2.67**	**0.009**	81.47**	61, 65, 66, 72, 95, 97, 114, 116, 138, 152, 157
	ABS	29	21.8 (6.4)	6.6 (2.7)	**3.12**	**0.002**		
	Non-ABS	153	2.7 (0.7)	1.6 (0.6)	1.17	0.245		

N is the number of codons in each category. The *d*_N_ and *d*_S_ values are given as the percentage per site. The standard errors in parentheses were computed using 1000 bootstrap replicates. The inferred positively selected codons located at ABSs are underlined. Significant results are highlighted in bold.﻿﻿ **p < 0.01

Screening for recombination with RDP yielded two DLA-88 alleles as intralocus recombinants, with one DLA-64 allele and one DLA-79 allele as interlocus recombinants (Additional file 6: Fig. S2). Analysis using GARD identified two significant breakpoints (268, 444; *p* < 0.01), and the first breakpoint was located at the boundary of exon 2 and exon 3 (Additional file 6: Fig. S2). RDP4 or the GARD method did not provide significant evidence of intralocus recombination within the other three MHC I genes. These combined findings suggested that positive selection and recombination (intergenic/intragenic recombination) have shaped the evolutionary patterns of the MHC I genes of the wolf.

### Signatures of positive selection against a background of purifying selection across the carnivorous TLR gene family

By combining assembled unigenes and clone-sequencing results, we obtained the full CDS of 10 TLRs in the wolf [GenBank: KX756657-KX756666]. Comparison of the ratio of *d*_N_ and *d*_S_ revealed distinct overall purifying selection for all TLRs across the carnivorous lineage, with mean ω values ranging from 0.173 to 0.527 (Table 4).

**Table 4:** Positively selected sites across carnivorous TLRs as inferred by different methods

Locus	Size (aa)	-2lnΔL	PAML M8	FEL	REL	Total No. of sites	Sites located in LRRs	ω
TLR1	789	11.57**	629	69, 289, 291, 316, 629	289, 291, 316, 324, 326, 366, 395, 561, 629	10	8	0.527
TLR2	766	5.88	None	557, 609	487, 557, 609	3	1	0.348
TLR3	905	2.02	None	13, 270, 293	13, 69, 270, 279, 293, 344, 587	7	6	0.307
TLR4	833	7.24*	193, 295, 341	46, 193, 319, 562, 564	46, 58, 91, 139, 193, 295, 306, 308, 319, 321, 339, 341, 347, 364, 369, 384, 393, 394, 464, 505, 510, 514, 517, 562, 564, 620, 832	27	24	0.493
TLR5	858	1.60	None	63, 137, 609	None	3	2	0.252
TLR6	797	2.34	None	109, 631	None	2	1	0.491
TLR7	1050	N.A.	None	None	None	-	-	0.173
TLR8	1038	2.42	None	234, 457, 683, 689	202, 234, 390, 416, 457, 683, 689	7	7	0.226
TLR9	1032	10.70**	5	5, 457, 627	None	3	2	0.163
TLR10	807	N.A.	None	None	None	-	-	0.439

Positively selected codons inferred by more than one ML method are underlined. The overall ω for each TLR was calculated by the SLAC method. N.A. indicates not available because of a low -2lnΔL value. *p < 0.05, **p < 0.01

Among the ten TLRs, TLR3, TLR5, TLR7, TLR8 and TLR9 possessed lower ω values than did the other 5 TLRs. Interestingly, with the exception of TLR5, which presented an ω value of 0.252, the former TLR category corresponded to receptors known to recognize nucleic acids from viruses, thus indicating stronger purifying selection in viral TLRs. However, despite a global trend of purifying selection acting on TLRs in carnivores, evidence of statistically significant positive selection was detected across most loci via codon-based maximum likelihood (ML) methods (as indicated by the PAML M8, fixed-effect likelihood (FEL) and random effect likelihood (REL) methods), with a total of 62 codons (Table 4). Furthermore, 51 codons (82.3%) were observed in the extracellular LRR domain, thus suggesting that positive selection has acted mainly on pathogen recognition domains (Additional file 8: Tables S7–S16). The nature and strength of selection varied among 10 TLRs. For instance, the non-viral TLR4 exhibited a remarkable pattern of adaptive evolution, with 27 positively selected sites being identified. TLR7 appeared to show the strongest evolutionary constraint among the four virus TLRs, with no positively selected sites being identified by all three ML methods. We also noted that no codons under statistically significant positive selection were found in non-viral TLR10, although the ω value of this gene was greater than that of all virus TLRs (Table 4).

Species-specific positively selected sites were detected with the Mixed Effects Model of Evolution (MEME) algorithm. Eleven codon positions in 7 wolf TLRs (TLR2–6, TLR8–9) were found to be affected by episodic selection, and 7 of the TLRs were located directly in the LRRs. Within the 7 sites in LRRs, site 309 in TLR2, 491 in TLR3, 306 in TLR4, and 87 and 504 in TLR9 showed radical amino acid changes in terms of size, polarity, or electric charge (Additional file 9: Table S17).

## Discussion

### Overview of the wolf peripheral blood transcriptome

The gray wolf is one of the most widely distributed predators in the world. A recent study of wolves of different ecotypes has identified statistical signatures of selection in immune-related genes, thus indicating that the immune system has been involved in the adaptive evolution of the wolf to various habitats [34]. In this study, we established the genetic architecture of the wolf peripheral blood transcriptome to advance understanding of the wolf immunome, with a special focus on the adaptive evolution patterns of two pathogen recognition receptor gene families (MHC I and TLR). We obtained approximately 373 million high-quality clean reads and assembled them into 123,851 unigenes by using Trinity. The unigenes exhibited a maximum length of 18,148 bp and an N50 value of 1121 bp, and up to 83.7% clean reads mapped to the assembled unigenes, thus indicating a high assembly quality of transcriptome sequences.

We then compared the unigenes against public databases to perform further functional annotation and classification. GO annotation showed that cellular process and metabolic process were the most represented BP categories, in agreement with the results reported in research in pandas [12]. We also noted that 100 unigenes were annotated for antigen processing and presentation (Additional file 2: Table S1), representing a high level of genetic diversity in immune-related genes in the wolf. This result was consistent with recent studies of MHC genetic diversity in the wolf [20–22]. These heterogeneous genes involved in antigen processing and presentation may lead to the recognition of a wide spectrum of pathogens and epidemics in the wolf. Signal transduction and the immune system were the most common KEGG sub-categories, thus highlighting the primary functionality of intravascular blood in responding to dynamic environmental signals and in defenses against invading pathogens [8, 35]. Among the 15 identified immune pathways, the chemokine signaling pathway was the most represented, with all 5 known chemokine subfamilies being identified [36]. Interestingly, several studies have demonstrated that a range of genes in this pathway are involved in the wound healing process [37–39]. Consequently, it is reasonable to assume that the chemokine signaling pathway plays an important role in inflammation and host immune surveillance in the wolf, which is known to frequently suffer from a variety of injuries primarily resulting from scuffles with intended prey [40]. Pathways involving the MHC and TLR gene families were identified, thus suggesting that peripheral blood plays important roles in both the innate and adaptive immune systems. Furthermore, pathways functionally bridging the innate and adaptive immune responses were also identified (Table 2, Additional file 4: Table S3). These results revealed the complexity of immune mechanisms in wolf peripheral blood.

### MHC class I gene family

MHC genes constitute a central component of the adaptive immune response and are thought to be associated with pathogens resistance and the long-term survival of animals [15]. Therefore, research into this genetic system should provide valuable information about the underlying immune response mechanisms in animals. Although only 4 individuals were included in this study, we identified 17 alleles (exons 2–3) among the 4 MHC I genes. DLA-88 was found to be more polymorphic than the other three genes, in agreement with results from previous studies in dogs [23, 24]. DLA-12 also displayed a relatively high level of polymorphism, with 6 alleles identified. However, the variability of DLA-12 in dogs appears to be much more limited, and only 1 allele has been observed among 18 individuals [24]. Therefore, the DLA-12 polymorphic and genetic type may be related to the existent of divergent immunological properties between wolves and dogs. We also examined the expression levels of MHC genes and found that DLA-64 was the most highly expressed MHC gene (Additional file 7: Table S5). We successfully cloned DLA-12 and DLA-88 from wolf blood cDNA libraries, and the results were consistent with previous research on dogs [23, 41], thus suggesting that these two genes are expressed in canine blood. However, these two genes were not identified through the Nr BLAST results. We speculate that the abundant alleles and high sequence variability made it difficult to assemble the transcripts of DLA-12 and DLA-88, and quantitative real-time PCR may be a useful method for quantifying the expression levels of these two genes. Furthermore, wolf MHC class II genes, which were also expressed in wolf blood (Additional file 7: Table S5), showed greater polymorphism relative to that in other carnivores [21, 42]; this polymorphism may be necessary for wolves to cope with the complex pathogen environments of different habitats.

Previous studies have concluded that balancing selection maintains allelic diversity and an excess of *d*_N_ over *d*_S_ at MHC in vertebrates [43]. A higher value of ω at ABSs than non-ABSs supports the influence of balancing selection on the MHC I loci of the wolf, in accordance with published data on MHC I loci in other mammals [44–46]. Additionally, strong positive selection on ABSs has been reported for MHC II genes in wolves [21, 22]. Because the MHC genes are involved in antigen presentation, the increased number of nonsynonymous mutations in ABSs is likely to increase the wolf’s ability to recognize a diverse range of pathogens. Evidence of positive selection was detected in both DLA-12 and DLA-88, although the excess of nonsynonymous substitutions in the ABS of DLA-12 was not significant. Fifth codons were identified as positively selected sites by CODEML. Among these sites, 10 were located in ABSs, which are involved in peptide binding, thus suggesting that these sites may be of functional importance. Additionally, 1 and 7 positively selected sites were detected in the ABSs of DLA-12 and DLA-88 (Table 3, Additional file 6: Fig. S2), implying the existence of a conservative antigen-binding function for DLA-12 compared with DLA-88. In conclusion, different intensities of polymorphic and divergent selection might be indicative of distinct functional roles for wolf MHC I genes.

We detected clear evidence of intergenic/intragenic recombination that has shaped the evolution of wolf MHC I genes. An intragenic recombination effect was observed at DLA-88, but not at the other 3 MHC I loci, a result indicating the existence of distinct recombination patterns for different MHC I loci. Furthermore, we detected a significant breakpoint located near the boundary of exon 2 and exon 3 (Additional file 6:Fig. S2), which supports recombination through the mechanism of exon shuffling [47]. This mechanism has been identified in many lower vertebrates as well as several mammals, such as badgers and marmosets [48–50]. Collectively, our analyses supported a role of recombination (intra−/interlocus) and balancing selection in the generation and maintenance of wolf MHC I diversity. The complex evolutionary mechanisms of MHC I loci may indicate that the wolf has evolved its adaptive immunity along the history of host-pathogen coevolution.

### Toll-like receptor gene family

As a key family of innate immunity receptors, TLRs lie directly at the host-pathogen interface and might be involved in coevolutionary dynamics with their microbial counterparts [14]. This is the first study to thoroughly characterize the entire TLR repertoire of the wolf, and it provides the most extensive survey of TLR evolution in the carnivorous clade to date. We examined all 10 known TLRs and found a clear signature of purifying selection that was consistent with previous reports for primates and rodents [27, 28]. This conservative mode is presumably due to the need to preserve the well-established pathogenic sensing function of TLRs. We also found that viral TLRs were subject to stronger purifying selection than non-viral TLRs, except for TLR5. This dichotomy has been suggested to be due to the different nature of the microorganisms targeted by the two groups of TLRs [51]. Specifically, we suggest that the greater evolutionary flexibility of non-viral TLRs is compatible with more diverse PAMPs of non-viral pathogens. Among the 10 TLRs, TLR5 is the only member that recognizes an exclusively proteinaceous ligand of flagellins [52], and it is therefore reasonable to predict the existence of functional constraint for TLR5. Interestingly, TLR5 was the TLR under the strongest purifying selection among non-viral TLRs (ω = 0.252), a pattern essentially the same as that reported in non-human primates [27]. In humans, however, TLR5 has been suggested to be functionally redundant. A loss-of-function TLR5 stop mutation is distributed nearly worldwide and exhibits a high frequency in some human populations (up to 23%) [53]. Another nonsynonymous mutation in human TLR5, leading to diminished NF-кB signaling, has recently been reported to confer a selective advantage [54]. Although the accessory mechanism of flagellin-recognition in the case of invalid TLR5 remains unclear, the different evolutionary pattern of TLR5 may reflect the different flagellin pathogen environments experienced by human and wildlife. TLR9 also appears to have evolved under strong stabilizing selection, as indicated by its lowest overall ω value. Furthermore, TLR9 is expressed at far higher levels than other TLRs (Additional file 7: Table S6). These results for TLR5 and TLR9 demonstrated their essential, non-redundant biological roles in carnivorous immune performance.

Despite pervasive purifying selection, we found evidence of positive selection acting upon a few codons, primarily within the pathogen recognition domains (i.e., LRRs) for most TLRs. This finding could be in agreement with species-specific differences in TLRs observed during related ligand recognition [55]. Carnivorous TLR4 displays a relatively high accumulation of codons exhibiting positive selection, a result consistent with those previously reported in other mammals [27, 28]. In addition to a well-established function in LPS recognition (i.e., the glycolipid in the outer membrane of Gram-negative bacteria), TLR4 has been implicated in the responses to other pathogens, including fungi, protozoans, and even viruses [56]. The broad spectra of targeted pathogens and the species-specific pathogenic environment could be the reasons for the remarkable pattern of adaptive evolution observed in TLR4. Further analysis has revealed that some of the sites (295, 339, 341 and 364) (Table 4) are located in or adjacent to the regions responsible for the interactions between TLR4 and LPS [57]. In particular, site 341, which was identified by both the PAML and REL methods, directly participates in the binding of LPS to TLR4 [57]. TLR1 acts in association with TLR2 in the recognition of microbial lipoproteins or lipopeptides [58] and was found to exhibit the second-highest number of positively selected codons. Several codons (289, 291, 316, 324, 326 and 366) (Table 4) under selection are located in LRRs that participate in interactions between TLR1, TLR2, and lipoproteins [58]. Two sites (316 and 326) were shown to directly participate in the binding of ligands to the TLR1-TLR2-lipopeptide complex, and two sites (324 and 366) were located directly at the dimerization interface between the TLR1 and TLR2 proteins [58]. With respect to TLR7 and TLR8, although they are thought to defense against most common viruses [26, 51], the selective pattern was discordant between these TLRs (Table 4). However, no positive selection codon was identified in TLR7, and the identified positively selected sites of TLR8 did not fall within functional ligand binding regions [59], thus suggesting functional constraint of these two genes among carnivores.

Documenting the genetic and selective landscape of TLRs can provide insights into the existing differences in infectious disease susceptibility and pathogenic resistance observed among different species or populations. For example, the signature of positive selection in the human TLR3 555I codon has been functionally characterized as mediating the recognition of the herpes simplex virus type-1 (HSV-1), which is naturally benign to humans, whereas non-human primates exhibit high susceptibility [60, 61]. In contrast, TLR4 D299G has been linked to different levels of susceptibility to bacterial infections as well as a higher prevalence of atopic asthma in Swedish children [62]. We used the MEME method in a wolf lineage to detect specific sites that showed positive selection, which can be missed by codon-based methods [63]. Overall, we detected 11 positively selected sites in 7 wolf TLRs, which may result in a broad spectrum of pathogen recognition in the wolf. Unfortunately, owing to the present insufficient understanding of the differences in susceptibility to infectious diseases between wolves and humans, it was not possible for us to associate these diseases with amino acid variations in wolf TLRs. However, some of the positively selected sites identified in this study were located in regions that participate in the binding of ligands to TLRs (Additional file 9: Table S17) and may affect pathogen recognition and defenses in the wolf.

## Conclusions

Utilizing Illumina sequencing technology and a genome-guided assembly strategy, we assembled and characterized the first peripheral blood transcriptome for the wolf. The wolf blood transcriptome provided a large-scale view of gene content for the characterization of wolf immunological properties and revealed an adaptive mechanism related to a complex pathogen environment. We found that many immune-related genes were expressed in wolf blood, and signal transduction and the immune system were the most represented KEGG functional groups. We also investigated the evolutionary patterns of two pathogen recognition gene families (MHC I and TLR), which might be involved in coevolutionary processes with pathogens. Balancing selection and recombination together have driven the historical evolution of wolf MHC I genes. Moreover, carnivorous TLRs have undergone adaptive evolution against a background of purifying selection, and a high level of adaptive evolution was detected in wolf TLR repertory. Finally, we advocate for further study of the evolutionary and selective landscape of immune-related genes of mammals to characterize the genetic mechanisms underlying differences in infectious disease susceptibility and pathogenic resistance observed among humans and wildlife.

## Methods

### Animals and samples collection

We collected blood samples from eight unrelated adult wolves from Tibet and Inner Mongolia in China. The habitat types of the two regions were desert plateau and steppe, respectively. Among the eight wolves, four individuals (one male and three females) originated from Tibet and were raised in Luobulingka Zoo in Tibet, and four individuals (two males and two females) came from Inner Mongolia, two of which were raised in the Dailake National Nature Reserve in Inner Mongolia, and two of which were raised in the Yantai Zoo in Shandong. Blood samples were collected from the leg vein of each wolf after the wolves were anesthetized. The fresh blood samples were immediately stored in RNAprotect® Animal Blood Tubes (QIAGEN, Germany) and frozen in liquid nitrogen until use. All experimental procedures were approved by the Qufu Normal University Institutional Animal Care and Use Committee (Permit Number: QFNU2015–005).

### RNA extraction and transcriptome sequencing

Total RNA was extracted using RNeasy® Protect Animal Blood Kit following the manufacturer’s protocol (QIAGEN, Germany). RNA purity was checked using the NanoPhotometer® spectrophotometer (IMPLEN, CA, USA). RNA concentration was assessed using Qubit® RNA Assay Kit in the Qubit® 2.0 Fluorometer (Life Technologies, CA, USA).

A total amount of 3 μg total RNA per sample was used as input material for the RNA sample preparations. Eight samples showed quality values with RNA integrity numbers between 8.0 and 9.2 as determined with the Bioanalyzer. Sequencing libraries were generated using NEBNext® Ultra™ RNA Library Prep Kit for Illumina® (NEB, USA) following manufacturer’s recommendations. At last, after purified using AMPure XP system, the library products were assessed on the Agilent Bioanalyzer 2100 system. The clustering of the index-coded samples was performed on a cBot Cluster Generation System using TruSeq SR Cluster Kit v3-cBot-HS (Illumia) according to the manufacturer’s instructions. After cluster generation, the prepared libraries were sequenced on an Illumina Hiseq 2500 platform and 125 bp paired-end reads were generated.

### Genome-guided de novo transcriptome assembly

Reads from eight wolves were combined to increase the accuracy and completeness of the assembly. Clean reads were obtained after removal of adapters and low-quality reads that might have negatively affected the analysis results using in-house Perl scripts. The retained reads were first aligned to the Ensembl dog reference genome (boxer genome, CanFam3.1) using TopHat v2.0.13 [64]. On the basis of the alignment results, the high-quality reads were used for genome-guided de novo transcriptome assembly using Trinity v2.1.1 [30, 65], with the parameter --genome_guided_bam. In this process, Trinity first portioned reads according to locus and then performed de novo transcriptome assembly at each locus. This strategy has been showen to be a powerful method for generating a high-quality transcriptome and covering genes missing from the genome [66, 67]. After assembly, the longest transcription sequence within the same locus was taken and defined as a unigene. FPKM [68] values were calculated using RSEM software [69] to quantify the unigenes.

### Functional annotation and classification of the blood transcriptome

All of the unigenes were used for BLAST searches with annotation against the Nr database (ftp://ftp.ncbi.nih.gov/blast/db/) using an E-value cut-off of 1E-5. Unigene sequences were also aligned and annotated by BLAST searches to other databases in the following order: Nt (ftp://ftp.ncbi.nih.gov/blast/db/), KOG (ftp://ftp.ncbi.nih.gov/pub/COG/KOG/), Swiss-Prot (http://www.uniprot.org/), KEGG (http://www.genome.jp/kegg/), and GO (http://www.geneontology.org/).

The results of BLAST searching against Nr database were imported into Blast2GO 3.2 [70] for GO term mapping. The outputs of GO mapping were submitted to the WEGO [71] for GO classification under three main domains: BP (Biological Process), CC (Cellular Component), and MF (Molecular Function). KEGG annotation was performed using the KOBAS 2.0 [72] based on BLAST results in KEGG database. Moreover, the unigenes were also aligned to the KOG database to predict and classify possible functions.

### Characterization of MHC I and TLR gene families

Previous studies and BLAST searches against the Nt database in this study have revealed that the 4 MHC I genes [23, 41] and 10 TLR genes are all expressed in canine blood. We used a cloning-sequencing method to obtain the above genes from blood complementary DNA (cDNA) libraries to improve sequence reliability. cDNA was synthesized using a PrimeScript™ Double Strand cDNA Synthesis Kit (TaKaRa, Dalian, China) according to the manufacturer’s instructions. For MHC I, the full-length sequences of exons 2–3 of 4 genes were amplified in 4 individuals with locus-specific primer sets. Full details of these primer sets are provided in Additional file 10: Table S18. The PCR products were purified with a QIAquick Gel Extraction kit (QIAGEN, Germany) and cloned using a pMD™18-T Vector Cloning kit (TaKaRa), according to the manufacturer’s instructions. A total of 21 clones for the highly polymorphic DLA-88 and 8–16 clones for the moderately polymorphic DLA-12, 64 and 79 were sequenced per individual to decrease the possibility of false homozygotes being detected as a result of amplification bias. We used the term “allele” for unique exon 2–3 variants of MHC I genes. Additionally, we recognized a clone sequence as an MHC I allele if it was detected in 2 independent PCR amplifications from a single individual or appeared in at least 2 individuals. To amplify the complete CDS of 10 TLRs, we used a series of overlapping primers designed on the basis of corresponding dog sequences in GenBank (http://www.ncbi.nlm.nih.gov/genbank/) and the assembled related unigenes. The PCR primers for the 10 TLRs are presented in Additional file 11: Table S19.

### Analysis of adaptive evolution in the MHC I and TLR gene families

The obtained sequences from the two gene families were examined and assembled using DNASTAR4 (http://www.dnastar.com/). Nucleotide sequence alignment and amino acid translation were performed using MEGA v5.0 [73].

For the MHC I genes, we used the modified Nei–Gojobori method with the Jukes-Cantor correction to compute the average *d*_S_ and *d*_N_ at all codons, ABSs and non-ABSs. Significant differences in the *d*_N_/*d*_S_ ratio were determined with a codon-based Z-test after 1000 bootstrap replicates in MEGA (test statistic: Z). The putative ABS locations of DLA genes were inferred from information of human MHC I genes [74]. Because the ABS positions in 3 human class I genes (HLA, B, C) are slightly different, we conservatively treated the consensus ABSs of the 3 HLA genes as the ABSs of the DLA genes. Moreover, the site model implemented in CODEML from PAML v4.8 [75] was used to perform analyses of selective pressure at individual codons for MHC I genes. Two alternative models were implemented in CODEML, one of which (M7) restricted sites to neutral evolution or evolution under purifying selection (ω ≤ 1), whereas M8 allowed a class of sites to evolve under positive selection (ω > 1). A likelihood ratio test (LRT) with 2 degrees of freedom was then applied to compare the two nested models, and the codons under positive selection were identified using the Bayes Empirical Bayes approach (BEB), with a posterior probability higher than 95%. The recombination signal was tested by using seven algorithms (RDP [76], GENECONV [77], Chimaera [78], MaxChi [79], BootScan [80], SiScan [81], and 3Seq [82]) implemented in the Recombination Detection Program version 4 (RDP4) program suite [83] with default settings. Moreover, the online GARD tool [84] from the Datamonkey web server (http://www.datamonkey.org/) was used for assessing the presence of historical recombination signals.

Other carnivorous TLR sequences used in this study were retrieved from GenBank and Ensembl. For each TLR subset, 6–9 species from some of the most representative families of carnivores (Canidae, Ursidae, Odobenidae, Phocidae, Mustelidae and Felidae) were used. Odobenidae and Phocidae were not included in the TLR5 and TLR9 subsets, owing to the lack of data. Detailed species and sequence accession numbers are given in Additional file 12: Table S20. Selective pressure analyses at individual codons of each TLR were first performed by using the M7 and M8 models in CODEML. All sequences of each TLR were then analyzed through two other ML methods (FEL and REL), which were implemented in the HyPhy package [85] available from the Datamonkey server. For these analyses, we used the automatic model selection tool on the server to determine the best-fitting nucleotide substitution model. Codons with *p* values <0.1 for FEL and a Bayes Factor > 50 for REL were considered candidates for selection. In addition, the single likelihood ancestor counting (SLAC) method was used to obtain the overall ω for each TLR.

The imprint of natural selection on a functional gene is often difficult to detect because selection is frequently transient and episodic (i.e., it affects only a subset of lineages) [63]. We subsequently searched for evidence of episodic diversifying selection at individual sites along the branches of the trees by using the powerful MEME method on the Datamonkey server. This method allows the ω value to vary among sites along branches in the tree and can be used to detect positive selection that may occur only in restricted lineages. We primarily focused our attention on positively selected sites detected in the wolf branch, and the default α = 0.1 was used as the significance threshold for MEME. Ancestral amino acid sequences were reconstructed via a Bayesian method using ANCESTOR [86], and the amino acid changes at positively selected sites were then determined on the basis of ancestral and extant amino acid sequences. We assessed the functional significance of positively selected sites by mapping these sites to the structural domains of each protein, which were predicted using the software LRRfinder [87] with upper and lower boundaries fixed at 95% and 80%, respectively.

## Additional files

Additional file 1: Fig. S1. Transcriptome assembly of wolf blood BLAST statistical analysis. (DOCX 188 kb)

Additional file 2: Table S1. GO functional classification of wolf blood transcriptome. (XLSX 29 kb)

Additional file 3: Table S2. KEGG functional classification of wolf blood transcriptome. (XLSX 159 kb)

Additional file 4: Table S3. Immune-related KEGG pathways in wolf blood transcriptome. (XLSX 25 kb)

Additional file 5: Table S4. KOG functional classification of wolf blood transcriptome. (XLSX 103 kb)

Additional file 6: Fig. S2. Multiple sequence alignments of the deduced amino acid sequences for exon 2–3 of the wolf MHC I genes. (DOCX 808 kb)

Additional file 7: Table S5–S6. MHC and TLR genes identified in the assembled unigenes. (XLSX 19 kb)

Additional file 8: Table S7–S16. Domain characterization of wolf TLR1–10 determined by LRRfinder. (DOCX 54 kb)

Additional file 9: Table S17. Species-specific positive selection sites in wolf TLR repertoire by MEME method. (DOCX 21 kb)

Additional file 10: Table S18. Wolf MHC I primer sets. (DOCX 19 kb)

Additional file 11: Table S19. Wolf TLRs primer sets. (XLSX 28 kb)

Additional file 12: Table S20. Identification of the sequences used for each TLR alignment. (XLSX 10 kb)
