# Supplementary material for: Characterization of the peripheral blood transcriptome and adaptive evolution of the MHC I and TLR gene families in the wolf (Canis lupus)
Source: BMC Genomics. 2017 Aug 7;18:584. doi: 10.1186/s12864-017-3983-0 (PMC5545864; doi:10.1186/s12864-017-3983-0)

Additional file 1: Figure S1. Transcriptome assembly of wolf blood BLAST statistical analysis. Venn diagram showing distribution of matches against 5 public protein databases (A). Distribution of species taxonomy (B), E-value distribution (C), and Identity distribution (D) by BLAST searches against the Nr database.


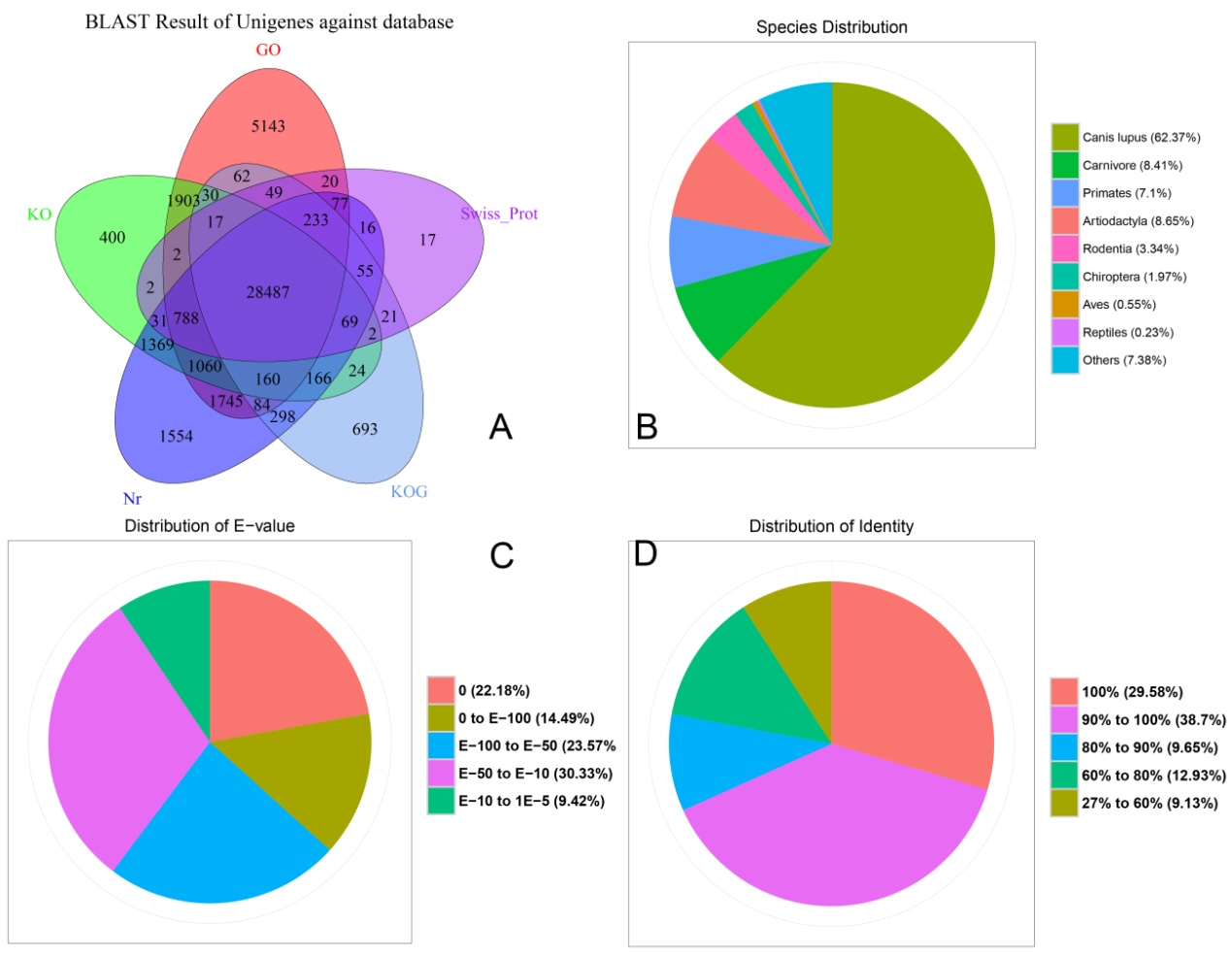

Supplement: Supplementary file 1 — Transcriptome assembly of wolf blood BLAST statistical analysis. (DOCX 188 kb) [file 12864_2017_3983_MOESM1_ESM.docx]
