# Supplementary material for: Characterization of the peripheral blood transcriptome and adaptive evolution of the MHC I and TLR gene families in the wolf (Canis lupus)
Source: BMC Genomics. 2017 Aug 7;18:584. doi: 10.1186/s12864-017-3983-0 (PMC5545864; doi:10.1186/s12864-017-3983-0)

Additional file 6: Figure S2. Multiple sequence alignments of the deduced amino acid sequences for exon 2-3 of the wolf MHC I genes. Dots indicate identities and hyphens represent amino acids gaps to the other sequences. Crosses represent putative ABS (antigen-binding sites) inferred from the human MHC I genes (HLA, -B, -C). The boxed crosses represent putative ABS under positive selection identified by PAML M8. Red arrows represent breakpoints identified by GARD method. Star and circle symbols represent the detected intralocus and interlocus recombinants, respectively.


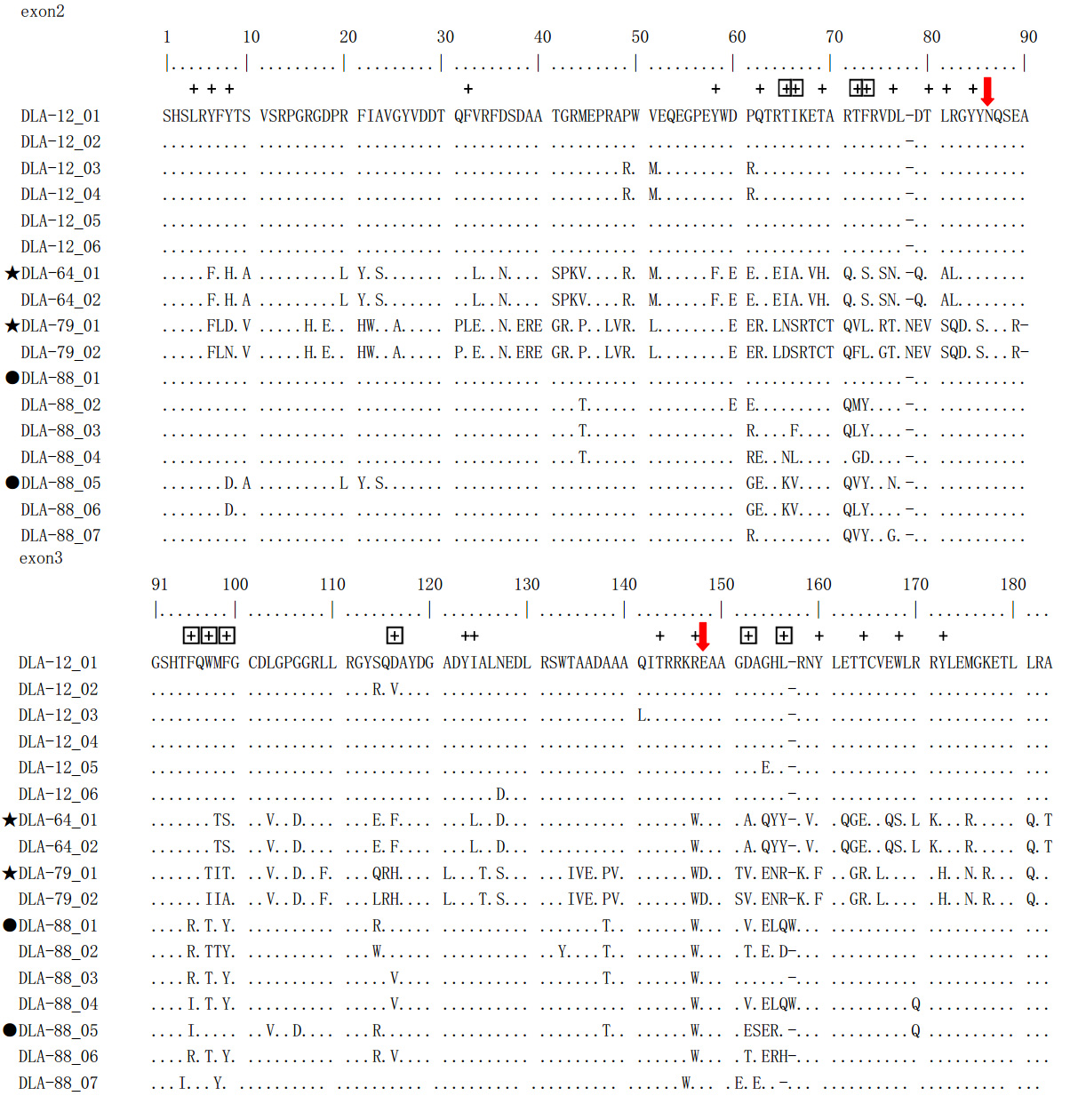

Supplement: Supplementary file 6 — Multiple sequence alignments of the deduced amino acid sequences for exon 2–3 of the wolf MHC I genes. (DOCX 808 kb) [file 12864_2017_3983_MOESM6_ESM.docx]
