# Supplementary material for: Characterization of the peripheral blood transcriptome and adaptive evolution of the MHC I and TLR gene families in the wolf (Canis lupus)
Source: BMC Genomics. 2017 Aug 7;18:584. doi: 10.1186/s12864-017-3983-0 (PMC5545864; doi:10.1186/s12864-017-3983-0)
Supplement: Supplementary file 8 — Domain characterization of wolf TLR1–10 determined by LRRfinder. (DOCX 54 kb) [file 12864_2017_3983_MOESM8_ESM.docx]

**Table S7. Domain characterization of TLR1.**

The conserved amino acid sequence of each LRR is shadowed. The amino acids identified as positive selection sites are boxed.

| **Domain** | **Start** | **Stop** | **Sequence** |
| --- | --- | --- | --- |
| Signal peptide | 1 | 25 | MKTNPSIFQFAIIFILILEIRIQLS |
| LRR-NT | 26 | 49 | EESDFLVNRSKAGLFHIPKDLSLK |
| LRR-1 | 50 | 73 | TTILDISQNYISELQTSDILSLSK |
| 2 | 74 | 97 | LRILIVSYNRIQYLDISVFKFNQE |
| 3 | 98 | 118 | LEYLDLSHNELGRISCHPTVN |
| 4 | 119 | 143 | LKHLDLSFNAFDDLPICKEFGNMSQ |
| 5 | 144 | 166 | LEFLGLSATQLQKSSMLPIASLH |
| 6 | 167 | 191 | IRKVLLVLGDTYGKKEDPESLQKLN |
| 7 | 192 | 215 | TESLHIVFPIRKEFSFTLDVSVST |
| 8 | 216 | 248 | AVSLELSNIKCVPDGHGWSYFQNVLSKLQKNSR |
| 9 | 249 | 275 | LSSLTLNNIETTWNFFIMLLQLVWHTS |
| 10 | 276 | 301 | IEYFSISNVKLQGYPDFRDFDYSDTS |
| 11 | 302 | 330 | LKALSIHQVVSNAFNLPQSYIYKIFSNMN |
| 12 | 331 | 352 | IQNFTVSGTHMVHMVCPSQISP |
| 13 | 353 | 376 | FLHLDFSNNLLTDIVFKNCRNLIK |
| 14 | 377 | 402 | LETLSLQMNQLKELASIAQMTNEMKS |
| 15 | 403 | 427 | LQQLDISQNSLRYDENEGNCSWTRS |
| 16 | 428 | 449 | LLSLNMSSNILTDSVFRCLPPK |
| 17 | 450 | 472 | VKVLDLHDNRIRSIPKPIMKLED |
| 18 | 473 | 494 | LQELNVASNSLAHFPDCGTFNR |
| 19 | 495 | 518 | LSVLIIDSNSISNPSADFLQSCHN |
| 20 | 519 | 542 | IRSISAGNNPFQCTCELREFVQSL |
| LRR-CT | 519 | 580 | IRSISAGNNPFQCTCELREFVQSLGQVASKVVEGWPDSYKCDSPENYKGTLLKDFHVSPLSC |
| Transmembrane | 581 | 607 | NTTLLLVTIGVAVLVFTVTVTALCIYF |
| TIR | 608 | 789 | DLPWYLRMVFQWTQTRRRARNTPLENLQRTIQFHAFISYSGHDSAWVKSELLPNLEKEELRICLHERNFIPGKSIVENIINCIEKSYKSIFVLSPNFVQSEWCHYELYFAHHNLFHEGSNNLILILLEPIPQYSIPSSYHKLKNLMAQRTYLEWPKEKSKHGLFWANLRASINIKLREQAKK |

**Table S8. Domain characterization of TLR2.**

The conserved amino acid sequence of each LRR is shadowed. The amino acids identified as positive selection sites are boxed.

| **Domain** | **Start** | **Stop** | **Sequence** |
| --- | --- | --- | --- |
| Signal peptide | 1 | 17 | MSRVLWTLWVLGAVTNL |
| LRR-NT | 18 | 54 | SKEEAPDQSSSLSCDPTGVCDGRSRSLNSMPSGLTAA |
| 1 | 55 | 78 | VRSLDLSNNEITYIGNSDLRDCVN |
| 2 | 79 | 102 | LKALRLESNGINTIEEESFFSLWS |
| 3 | 103 | 126 | LEHLDLSYNLLSNLSSSWFRPLSS |
| 4 | 127 | 151 | LKFLNLLGNPYKSLGETPLFSQLTN |
| 5 | 152 | 176 | LRILKVGNIYSFTEIQDKDFAGLTF |
| 6 | 177 | 200 | LEELEIDASNLQRYEPKSLKSIQN |
| 7 | 201 | 224 | ISYLALRMKQPVLLVEIFVDLSSS |
| 8 | 225 | 251 | LKHLELRDTHLDTFHFSEASINETHTL |
| 9 | 252 | 279 | VKKWTFRNVKVTDRSFTEVVRLLNYVSG |
| 10 | 280 | 309 | VLEVEFEDCTLYGLGDFDIPDVDKIKNIGQ |
| 11 | 310 | 338 | IETLTVRRLHIPHFYSFYDMSSIYSLTED |
| 12 | 339 | 362 | VKRITVESSKVFLVPCSLSQHLKS |
| 13 | 363 | 389 | LEYLDLSDNLMVEEYLRNSACQHAWPL |
| 14 | 390 | 415 | LQTLILRQNRLKSLEKTGETLLTLKN |
| 15 | 416 | 438 | LVNLDISKNNYLSMPETCQWPEK |
| 16 | 439 | 459 | LKCLNLSDTRMQSITRCIPQT |
| 17 | 460 | 479 | LEILDVSNNNLESFSLILPQ |
| 18 | 480 | 501 | LKELSISRNKLKTLPDASFLPT |
| 19 | 502 | 525 | LQIMRISRNTINAFSKEQLDSFHR |
| 20 | 526 | 549 | LQTLEAGGNNFLCSCEFLSFTQEQ |
| LRR-CT | 526 | 588 | LQTLEAGGNNFLCSCEFLSFTQEQQALAGLLVGWPEDYLCHSPSYVRGQRVGTARLPASECHR |
| Transmembrane | 589 | 611 | TALVAAVCCVLLLLVLLTAGACH |
| TIR | 612 | 785 | HFHGLWYLRMLWAWLQAKRKPRKAPSRDVCYDAFVSYSEHDSYWVENLLVQKLEHFNPPFKLCLHKRDFIPGKWIIDNIIDSIEKSHKTIFVLSENFVKSEWCKYELDFSHFRLFDENNDAAILILLEPIEKKAIPQRFCKLRKIMNTKTYLEWPTDDAQQEGFWLNLRTAIKS |

**Table S9. Domain characterization of TLR3.**

The conserved amino acid sequence of each LRR is shadowed. The amino acids identified as positive selection sites are boxed.

| **Domain** | **Start** | **Stop** | **Sequence** |
| --- | --- | --- | --- |
| Signal peptide | 1 | 25 | MSQSLLYHIYSFLGLLPFWILCTSS |
| LRR-NT | 26 | 53 | TNKCVVRHEVADCSHLKLTQVPDDLPAN |
| LRR1 | 54 | 77 | ITVLNLTHNQLRRLPPANFTRYSQ |
| LRR2 | 78 | 101 | LTILDGGFNSISKLEPELCQKLPL |
| LRR3 | 102 | 125 | LEILNLQHNELSHLSDQTFVFCVN |
| LRR4 | 126 | 149 | LTELHLMSNSIKIIQNNPFRSLKN |
| LRR5 | 150 | 173 | LVKLDLSHNGLSSTKLGSQLQLEN |
| LRR6 | 174 | 199 | LQELLLSNNKINVLRREELDFLGNSS |
| LRR7 | 200 | 223 | LEKLELSSNPIKEFSPGCFHAIGK |
| LRR8 | 224 | 250 | LFGLSLNNVQLNPSLTENLCLELSNTS |
| LRR9 | 251 | 276 | IQNLSLSNTQLHRTSNMTFLGLKHTN |
| LRR10 | 277 | 300 | LTMLDLSHNNLNVIENNSFVWLPH |
| LRR11 | 301 | 324 | LEYFLLEYNNIEHLFSHSFYGLLN |
| LRR12 | 325 | 357 | VRYLDLKRSFAKQSTSLASHPRIDDFSFQWLKC |
| LRR13 | 358 | 381 | LQYLNMEDNYFAGIKSNMFTGLIK |
| LRR14 | 382 | 409 | LKHLSLSNSFTSLQTLTNETFLSLAQSP |
| LRR15 | 410 | 433 | LITLNLTKNKISKIESGAFSWLGH |
| LRR16 | 434 | 458 | LQVLDLGLNEIGQELTGQEWRGLEN |
| LRR17 | 459 | 482 | IVEIYLSYNKYLQLTSSSFALIPS |
| LRR18 | 483 | 508 | LRRLMLRRTALRNVDSSPSPFHPLRN |
| LRR19 | 509 | 532 | LNILDLSNNNIANINDELLEGLEK |
| LRR20 | 533 | 564 | LEILDMQHNNLARLWKHANPGGPVHFLKGLSH |
| LRR21 | 565 | 588 | LHILNLESNGFDEIPAEVFKGLSE |
| LRR22 | 589 | 612 | LKSIDLGLNNLNIFPSSLFNDQVS |
| LRR23 | 613 | 637 | LKSLNLQKNLITSVEKNVFGPAFRN |
| LRR24 | 638 | 661 | LSNLDMSFNPFDCTCESIAWFVNW |
| LRR-CT | 638 | 703 | LSNLDMSFNPFDCTCESIAWFVNWINSTHTNISELSSHYLCNTPPQYHGFPVMLFDISPCKDSAPF |
| Transmembrane | 704 | 726 | EIFFIINTSVLLTFIFIVLLIHF |
| TIR | 727 | 905 | EGWRISFYWNVSVHRILGFKEIDKQPEQFEYAAYIIHAYKDRDWVWEHFSPMEEKDETLKFCLEERDFEAGVLELESIINSIKKSRKTIFVITQHLLKDPLCKRFKVHQAVQQAIEQNLESIILIFLEEIPDYKLNHALCLRRGMFKSHCILNWPVQKERVNAFHHKLQVALGSRNSIH |

**Table S10. Domain characterization of TLR4.**

The conserved amino acid sequence of each LRR is shadowed. The amino acids identified as positive selection sites are boxed.

| **Domain** | **Start** | **Stop** | **Sequence** |
| --- | --- | --- | --- |
| Signal peptide | 1 | 23 | MMSPTRLAGILIPAMAFLSCLRP |
| LRR-NT | 24 | 55 | ESWDPCMQVVANTTYQCMELNLSKIPNNIPTS |
| LRR1 | 56 | 79 | TEKLDLSFNPLRHLGSHCFSNFPK |
| LRR2 | 80 | 103 | LQVLDLSRCEIQVIEDDAYQGLNH |
| LRR3 | 104 | 127 | LSILILTGNPIQRLFPRAFSGLSS |
| LRR4 | 128 | 151 | LKTLVAKETKLTSLEDFPIGHLKT |
| LRR5 | 152 | 176 | LKELNVAHNLIHSFKLPAYFSNMPN |
| LRR6 | 177 | 204 | LENVDLSNNKIQNIYREDLQVLHQMPLL |
| LRR7 | 205 | 227 | NLSLDLSLNPLYFIQPGSFKEIK |
| LRR8 | 228 | 254 | LHELTLRSNFNSTDVMKTFIQGLAGLK |
| LRR9 | 255 | 284 | INQLVLGEFKNERKLESFDNSLLEGLCNLT |
| LRR10 | 285 | 309 | IEKFRIAYFDSFSKDTTNLFNQLVN |
| LRR11 | 310 | 331 | ISAISLAHLYLDTPKYLPKNLR |
| LRR12 | 332 | 352 | WQRLEIVNCNLEQFPAWELDS |
| LRR13 | 353 | 374 | LKEFVLTSNKGMNTFADMKMES |
| LRR14 | 375 | 400 | LEFLDLSRNRLSFKTCCSHSDFGTTR |
| LRR15 | 401 | 423 | LKHLDLSFNEIITMSSNFLGLEQ |
| LRR16 | 424 | 448 | LEYLDLQHSSLKQASDFSVFLSLRN |
| LRR17 | 449 | 472 | LRYLDISYTRTEVAFQGIFDGLVS |
| LRR18 | 473 | 497 | LEVLKMADNSFPDNSLPNIFKGLTN |
| LRR19 | 498 | 521 | LTILDLSRCHLERVSQESFVSLPK |
| LRR20 | 522 | 545 | LQVINMSHNSLLSLDTLAYEPLLS |
| LRR21 | 546 | 570 | LQILDCSFNRIVAFKEQGQQHFPSN |
| LRR22 | 571 | 594 | LVSLNLTRNNFACDCEHQSFLQWV |
| LRR-CT | 571 | 630 | LVSLNLTRNNFACDCEHQSFLQWVKDHRQLLVKVEQMVCAKPLDMKDMPLLSFRNATCQR |
| Transmembrane | 631 | 654 | SKTIISVSVFTVLMVSLVAVLAYK |
| TIR | 658 | 833 | HLMLLAGCKRYNRGESTYDAFVIYSSQDEDWVRNELVKNLEEGVPPFQLCLHYRDFIPGVAIAANIIQEGFYKSRKVIVVVSQHFIQSRWCIFEYEIAQTWQFLSSRAGIIFIVLQKVEKSLLRQQVELYRLLSRNTYLEWEDSVLGRHIFWRRLRKALLDGKPWSPEGTEDAEKS |

**Table S11. Domain characterization of TLR5.**

The conserved amino acid sequence of each LRR is shadowed. The amino acids identified as positive selection sites are boxed.

| **Domain** | **Start** | **Stop** | **Sequence** |
| --- | --- | --- | --- |
| Signal peptide | 1 | 20 | MGRQLGRTLGLLLVAGAVAA |
| LRR-NT | 21 | 47 | ASCCVADGRRALYRSCNLSQVPPVPST |
| LRR1 | 48 | 71 | TEILLLSFNYIRAVTRASFPLLER |
| LRR2 | 72 | 96 | LQLLELGTQQTPFSVDREAFRNLPN |
| LRR3 | 97 | 120 | LRTLDLGNSRVDFLHPDAFQGLPH |
| LRR4 | 121 | 146 | LQELRLFACGLSDVVLTDGYFRNLGA |
| LRR5 | 147 | 171 | LSRLDLSKNQIGSLELHASFRELGS |
| LRR6 | 172 | 197 | LRSVDFSLNRIPAACEQGLRPLQGKA |
| LRR7 | 198 | 227 | LSLLNLAANGLYSRAPVDWGRCGNPFRNVV |
| LRR8 | 228 | 254 | LETLDVSNNGWTADVTGNVTRAIGGSQ |
| LRR9 | 255 | 289 | ISSLVLAHHIMGQGFGFRNIRDPDRSTFAGLAGSS |
| LRR10 | 290 | 313 | VLRLDLSHGFVFSLNARLFEVLGD |
| LRR11 | 314 | 337 | LKLLDLAHNKINRIAGEAFHGLGS |
| LRR12 | 338 | 361 | VQVLNLSHNLLGELYDSDFSGLAE |
| LRR13 | 362 | 385 | VAYIDLQHNHIGIIQDQTFRFLGA |
| LRR14 | 386 | 404 | LRTLDLRDNALKTVSFVPS |
| LRR15 | 405 | 424 | IDTIFLGNNKLETVSHMDLT |
| LRR16 | 425 | 449 | ASFLELSDNRLEDLGDLYSLLRVPA |
| LRR17 | 450 | 474 | LQVLILNRNRLSACRGGHGPTGSVG |
| LRR18 | 475 | 503 | PERLFLGSNMLQLAWETGRCWDVFRGLPR |
| LRR19 | 504 | 527 | LRVLHLNHNYLAALPPGLLRDLTA |
| LRR20 | 528 | 549 | LRGLDLSANRLSTLSRGDLPAA |
| LRR21 | 550 | 570 | LEVLDVSRNQLLSLDPGLLAP |
| LRR22 | 571 | 594 | LRAVDLTHNKFICGCELRPLVRWL |
| LRR-CT | 571 | 637 | LRAVDLTHNKFICGCELRPLVRWLNRTNVTVFGSRADVRCAYPSSLAGTPLSSVSMEGCDDEEALRT |
| Transmembrane | 644 | 666 | IFSTVGVTLFLLAVLVATKLRGL |
| TIR | 667 | 858 | CFLCYKAARRLLPAGPAEDGAPDAYQYDAYLCFSGRDFEWVQRALLRHLDAQYSSRNRLNLCFEERDFVPGREHIANIQDAVWSSRKVVCLVSRHFLRDGWCLEAFAAARSRCASHLDGALVLVVVGSLSQYQLRRHPAIGGFVRQRQYLRWPEDLQDVGWFLDTLSRHILQEQRGARGDGGIPLRTVAAVA |

**Table S12. Domain characterization of TLR6.**

The conserved amino acid sequence of each LRR is shadowed. The amino acids identified as positive selection sites are boxed.

| **Domain** | **Start** | **Stop** | **Sequence** |
| --- | --- | --- | --- |
| Signal peptide | 1 | 23 | MIKDKDSITGSFHFVYIVTLIVG |
| LRR-NT | 24 | 53 | TIIQFSDESEFTVDMSNMNLTHVPEDLPPK |
| LRR1 | 54 | 77 | TKILDMSQNNISELHLSDMSYLSG |
| LRR2 | 78 | 101 | LKILRISHNRIWWLDFSIFKFNQD |
| LRR3 | 102 | 122 | LEYLDLSYNQLRNMSCHLIRS |
| LRR4 | 123 | 147 | LKHLDLSFNDFHVLPICKEFGNLTQ |
| LRR5 | 148 | 170 | LQFLGLSATKLRQLDLLPIAHLH |
| LRR6 | 171 | 195 | LSYILLDLQGYYAKESEKGSLQILD |
| LRR7 | 196 | 219 | TKTLHLVFHPNQLFSVQANMLVNN |
| LRR8 | 220 | 250 | LGCLQLTNIKLNNDNCQVLIQFLSELTRGPT |
| LRR9 | 251 | 277 | LLNFTLQHVKTTWKCLVRIFKFLWPKP |
| LRR10 | 278 | 303 | VQYLNIYNLTIVESINKEYIHYPKTA |
| LRR11 | 304 | 332 | LKALTIEHVKNEVFLFSQTALYTIFSEMN |
| LRR12 | 333 | 354 | IMMLTISDTPFIHMLCPPPSNT |
| LRR13 | 355 | 378 | FKFLNFTQNVFTDSVFQSCSHLVR |
| LRR14 | 379 | 404 | LETLILRKNKLKDLYKVGLMTKHMTS |
| LRR15 | 405 | 429 | LEILDVSVNSLEYDRYDGNCTWVGS |
| LRR16 | 430 | 451 | IVVLNLSSNILTDSVFRCLPPK |
| LRR17 | 452 | 474 | VKVLDLHDNRIRSIPKPIMKLED |
| LRR18 | 475 | 496 | LQELNVASNSLAHFPDCGTFNR |
| LRR19 | 497 | 520 | LSVLIIDSNSISNPSADFLQSCHN |
| LRR20 | 521 | 544 | IRSMSAGNNPFQCTCELREFVQSL |
| LRR-CT | 521 | 584 | IRSMSAGNNPFQCTCELREFVQSLGQVASKVVEGWPDSYKCDSPENYKGTLLKDFHVSPLSCNT |
| Transmembrane | 585 | 609 | TLLLVTIGVAVLVFTVTVTALCIYF |
| TIR | 610 | 797 | DLPWYLRMVFQWTQTRRRARNTPLEELQRTIQFHAFISYSEHDSAWVKNELVPCLEKEELRICLHERNFIPGKSIVENIINCIEKSYKSIFVLSPNFVQSEWCHYELYFAHHNLFHEGSNNLILILLEPIPQNCIPSKYHKLRALMTQRTYLEWPKEKSKHGLFWANIRAAFNMKLTLIAENNNAEAS |

**Table S13. Domain characterization of TLR7.**

The conserved amino acid sequence of each LRR is shadowed. The amino acids identified as positive selection sites are boxed.

| **Domain** | **Start** | **Stop** | **Sequence** |
| --- | --- | --- | --- |
| Signal peptide | 1 | 26 | MVFPMWTLKRQFFILLNIILISKLLG |
| LRR-NT | 27 | 66 | ARWFPKTLPCDVSLDAPKAHVIVDCTDKHLTEIPGGIPSN |
| LRR1 | 67 | 90 | ATNLTLTINHIPGISPASFHQLDY |
| LRR2 | 91 | 128 | LVEIDFRCNCIPVRLGPKDHLCTRRPQIKPRSFSSLTY |
| LRR3 | 129 | 149 | LKSLYLDGNQLLEIPEGLPPS |
| LRR4 | 150 | 173 | LELLSLEANSIFSIMKNNLTELTN |
| LRR5 | 174 | 205 | IERLYLGQNCYFRNPCNVSFFIEKDAFLSLKN |
| LRR6 | 206 | 226 | LKLLSLKDNNITYVPTTLPST |
| LRR7 | 227 | 250 | LTELYLYNNAIAKIQEDDFNNLNQ |
| LRR8 | 251 | 291 | LRILDLSGNCPRCYNVPFPCTPCENNSPLQIHESAFDALTE |
| LRR9 | 292 | 315 | LQVLRLHSNSLQRVPQRWFKNIKK |
| LRR10 | 316 | 341 | LKELDLSQNFLAKEIGDAKFLYLLHD |
| LRR11 | 342 | 371 | LVQLDLSFNYELQVYRAALNLSDAFSSLKN |
| LRR12 | 372 | 398 | LKVLRIKGYVFKELSSHHLSPLQSLTN |
| LRR13 | 399 | 422 | LEVLDLGTNFIKIADLSIFEQFKT |
| LRR14 | 423 | 495 | LKVIDLSMNKISPSGDSGEVGFCSSTRTSVEGHAPQVLETLHYFRYDEYARSCRFKNKETPSFLPFNKDCYMY |
| LRR15 | 496 | 519 | GQTLDLSRNNIFFIKSSDFQHLSF |
| LRR16 | 520 | 544 | LKCLNLSGNTIGQTLNGSEFQPLVE |
| LRR17 | 545 | 568 | LKYLDFSNNRLDLLYSTAFEELRK |
| LRR18 | 569 | 598 | LEVLDISSNSHYFQSEGITHMLNFTKNLKV |
| LRR19 | 599 | 621 | LKKLMMNNNDIATSTSRTMESES |
| LRR20 | 622 | 652 | LKILEFRGNHLDVLWRDGDNRYLKFFKNLLN |
| LRR21 | 653 | 677 | LEELDISENSLSFLPSGVFDGMPPN |
| LRR22 | 678 | 701 | LKTLSLVKNGLKSFHWERLQYLKN |
| LRR23 | 702 | 725 | LETLDLSYNELKIVPERLYNCSRS |
| LRR24 | 726 | 749 | LKKLILKYNQIRQLTKHFLQDAFQ |
| LRR25 | 750 | 775 | LRYLDLSSNKIQIIQKTSFPENVLNN |
| LRR26 | 776 | 799 | LEMLLLHHNRFLCTCDAVWFVWWV |
| LRR-CT | 776 | 838 | LEMLLLHHNRFLCTCDAVWFVWWVNHTEVTIPYLATDVTCVGPGAHKGQSVVSLDLYTCELDL |
| Transmembrane | 839 | 863 | TNLVLFSFSLSLALFLMVITTANH |
| TIR | 894 | 1050 | AFVVYDTKDPAVTEWVLDELVAKLEDPREKHFNLCLEERDWLPGQPVLENLSQSIQLSKKTVFVMTNKYAKTENFKIAFYLSHQRLMDEKVDVIILIFLEKLLQKSKFLQLRKRLCKSSVLEWPRNPQAHPYFWQCLKNALATDNHVTYSQVFKETV |

**Table S14. Domain characterization of TLR8.**

The conserved amino acid sequence of each LRR is shadowed. The amino acids identified as positive selection sites are boxed.

| **Domain** | **Start** | **Stop** | **Sequence** |
| --- | --- | --- | --- |
| Signal peptide | 1 | 18 | MSPRSLVLTCLFLLISDS |
| LRR-NT | 19 | 61 | YEFVTKANYSRSYPCDERRQNGSVIAECNGRRLQEVPQTVGKY |
| LRR1 | 62 | 85 | VTVLDLSDNYITHITNESFHGLQN |
| LRR2 | 86 | 122 | LTKINLNHNANPQHLSENPDNKNGMNITDGAFLNLQN |
| LRR3 | 123 | 143 | LNQLLLEDNQLYQIPAGLPGS |
| LRR4 | 144 | 167 | LKELSLIQNNIIWVTKKNTSGLTN |
| LRR5 | 168 | 200 | LERLYLSWNCYFGNNCNNKTFNIEDGTFESLTN |
| LRR6 | 201 | 221 | LEVLSLSFNKLVHVPPKLPSS |
| LRR7 | 222 | 245 | LKELYLSNAKIKIISQEDFKGLRN |
| LRR8 | 246 | 286 | LRVLDLSGNCPRCFNAPFPCTPCEGSASIQIHPLAFQTLTE |
| LRR9 | 287 | 310 | LRYLNLSSTSLRKIPATWFDNMRN |
| LRR10 | 311 | 336 | LKVLHLEFNYLVDEIASGEFLTKLPV |
| LRR11 | 337 | 366 | LEILDLSYNYVKAKYPKYINISHNFSSLKL |
| LRR12 | 367 | 393 | LQALHLRGYVFQELRAGDFEPLMGLSN |
| LRR13 | 394 | 417 | LKTINLGVNFIKQINFTLFQNFPN |
| LRR14 | 418 | 480 | LSIIYLSENRISPLVNDIRQNEVNGSSSQRHVLKPRSADMEFDPHSNFYHNTHPLIKPQCTVY |
| LRR15 | 481 | 504 | GKALDLSLNSIFFIGREQFEAFHD |
| LRR16 | 505 | 529 | IACLNLSSNGNGQVLHGNEFSAVPH |
| LRR17 | 530 | 553 | IKYLDLTNNRLDFDDDNALSDLPE |
| LRR18 | 554 | 583 | LEVLDLSYNAHYFRIAGVTHRLGFIQNLTQ |
| LRR19 | 584 | 606 | LKVLNLSHNSIYTLTEQDLRSVS |
| LRR20 | 607 | 637 | LEELVFSGNRLDILWNAEGDKYWKIFTRLRN |
| LRR21 | 638 | 662 | LTRLDLSLNNLRRIPNEAFLNLPQS |
| LRR22 | 663 | 686 | LTQLYIKNNALNFFNWTLLQEFPR |
| LRR23 | 687 | 710 | LQVLDLSGNRLSSITNSLSKFTSS |
| LRR24 | 711 | 734 | LQTLLLHRNRISHLPASFLSEASS |
| LRR25 | 735 | 760 | LIHLDLSSNLLKMINKSTLQTKTNTS |
| LRR26 | 761 | 784 | LAILELGRNPFDCTCDIGDFRRWM |
| LRR-CT | 761 | 822 | LAILELGRNPFDCTCDIGDFRRWMDENLNVTIPRLTDVICSSPGDQRGKSIVSLELTTCISD |
| Transmembrane | 823 | 845 | TLAAVLCIFTSFITVTVMLAALG |
| TIR | 846 | 1038 | HHWFYWDVWFIYHVCLAKVKGYRSVSTSQTFYDAYVSYDTKDASVTDWVINELRFHLEESEGKNVLLCLEERDWDPGLAIIDNLMQSINQSKKTIFVLTKEYAQNWNFKTAFYLALQRLMDENMDVIIFILLEPVLQHSQYLRLRQRICKSSILQWPDNPKAEGLFWQSLKNVVLTENDSRYNNLYVDSIKQY |

**Table S15. Domain characterization of TLR9.**

The conserved amino acid sequence of each LRR is shadowed. The amino acids identified as positive selection sites are boxed.

| **Domain** | **Start** | **Stop** | **Sequence** |
| --- | --- | --- | --- |
| Signal | 1 | 25 | MGPCRGALHPLSLLVQAAVLALALA |
| Signal peptide | 26 | 64 | QGTLPAFLPCELQPHGLVNCNWLFLKSVPRFSAAAPRGN |
| LRR1 | 65 | 88 | VTSLSLYSNRIHHLHDYDFVHFVH |
| LRR2 | 89 | 124 | LRRLNLKWNCPPASLSPMHFPCHMTIEPNTFLAVPT |
| LRR3 | 125 | 144 | LEDLNLSYNSITTVPALPSS |
| LRR4 | 145 | 168 | LVSLSLSRTNILVLDPATLAGLYA |
| LRR5 | 169 | 200 | LRFLFLDGNCYYKNPCQQALQVAPGALLGLGN |
| LRR6 | 201 | 221 | LTHLSLKYNNLTVVPRGLPPS |
| LRR7 | 222 | 245 | LEYLLLSYNHIITLAPEDLANLTA |
| LRR8 | 246 | 285 | LRVLDVGGNCRRCDHARNPCRECPKGFPQLHPNTFGHLSH |
| LRR9 | 286 | 309 | LEGLVLRDSSLYSLDPRWFHGLGN |
| LRR10 | 310 | 335 | LMVLDLSENFLYDCITKTKAFYGLAR |
| LRR11 | 336 | 365 | LRRLNLSFNYHKKVSFAHLHLASSFGSLLS |
| LRR12 | 366 | 392 | LQELDIHGIFFRSLSETTLQSLAHLPM |
| LRR13 | 393 | 416 | LQRLHLQLNFISQAQLSIFGAFPG |
| LRR14 | 417 | 475 | LRYVDLSDNRISGAAEPAAATGEVEADCGERVWPQSRDLALGPLGTPGSEAFMPSCRTL |
| LRR15 | 476 | 499 | NFTLDLSRNNLVTVQPEMFVRLAR |
| LRR16 | 500 | 524 | LQCLGLSHNSISQAVNGSQFVPLSN |
| LRR17 | 525 | 548 | LRVLDLSHNKLDLYHGRSFTELPR |
| LRR18 | 549 | 578 | LEALDLSYNSQPFSMRGVGHNLSFVAQLPA |
| LRR19 | 579 | 601 | LRYLSLAHNGIHSRVSQQLRSAS |
| LRR20 | 602 | 631 | LRALDFSGNTLSQMWAEGDLYLRFFQGLRS |
| LRR21 | 632 | 656 | LVQLDLSQNRLHTLLPRNLDNLPKS |
| LRR22 | 657 | 680 | LRLLRLRDNYLAFFNWSSLALLPK |
| LRR23 | 681 | 704 | LEALDLAGNQLKALSNGSLPNGTQ |
| LRR24 | 705 | 728 | LQRLDLSGNSIGFVVPSFFALAVR |
| LRR25 | 729 | 753 | LRELNLSANALKTVEPSWFGSLAGA |
| LRR26 | 754 | 777 | LKVLDVTANPLHCACGATFVDFLL |
| LRR-CT | 754 | 814 | LKVLDVTANPLHCACGATFVDFLLEVQAAVPGLPSRVKCGSPGQLQGRSIFAQDLRLCLDE |
| Transmembrane | 815 | 835 | ALSWVCFSLSLLAVALSLAVPML |
| TIR | 836 | 1032 | MLHQLCGWDLWYCFHLCLAWLPRRGRRRGVDALAYDAFVVFDKAQSSVADWVYNELRVQLEERRGRRALRLCLEERDWVPGKTLFENLWASVYSSRKTLFVLARTDRVSGLLRASFLLAQQRLLEDRKDVVVLVILCPDAHRSRYVRLRQRLCRQSVLLWPHQPSGQRSFWAQLGTALTRDNRHFYNQNFCRGPTTA |

**Table S16. Domain characterization of TLR10.**

The conserved amino acid sequence of each LRR is shadowed. The amino acids identified as positive selection sites are boxed.

| **Domain** | **Start** | **Stop** | **Sequence** |
| --- | --- | --- | --- |
| Signal peptide | 1 | 21 | MKRIRSIYIFCSIAISVQGWA |
| LRR-NT | 22 | 49 | SKLPEERELTTNCSNMSLRKIPADLTPT |
| LRR1 | 50 | 73 | TTTLDLSYNLLSQLQSSDFRSVSK |
| LRR2 | 74 | 97 | LKVLILCHNRIQELNIKIFEFNRE |
| LRR3 | 98 | 118 | LRYLDLSYNRLKIVTWYSLAG |
| LRR4 | 119 | 143 | LRHLDLSFNDFDTVPICEETGNMSH |
| LRR5 | 144 | 166 | LEILGLSGAKIQKSNFQKIAHLH |
| LRR6 | 167 | 189 | LKTVFLGLRSLSHYEEGSLPILN |
| LRR7 | 190 | 213 | TTKLHIVLPMNTNFWVLLHDGIKT |
| LRR8 | 214 | 243 | SKILEMTNIDGKSQFASYGTQKNLTLENSK |
| LRR9 | 244 | 270 | TSILLLNKVDLLWDDLLLIFQFVWHTS |
| LRR10 | 271 | 298 | VECFQIQHLTFGGKVYLDHYSFDYSNTV |
| LRR11 | 299 | 327 | MRTIKLEHVQFRIFYIPQERVYLLFTKMD |
| LRR12 | 328 | 349 | IENLTISDAQMPYMLFPIYPTR |
| LRR13 | 350 | 373 | FQYLNFANNILTDDLFKQPIQLPH |
| LRR14 | 374 | 398 | LKTLILKGNKLETFSLVSFFANNTS |
| LRR15 | 399 | 422 | LKHLDLSQNLLQHENGENCFWPET |
| LRR16 | 423 | 444 | LITMNLSSNKFADSVFRCLPRN |
| LRR17 | 445 | 467 | IQILDLNNNKIQTVPKDIIHLKS |
| LRR18 | 468 | 489 | LQELNLAFNFLTDLPGCSHFRK |
| LRR19 | 490 | 513 | LSILNIEMNLILSPSLDFFQSCQE |
| LRR20 | 514 | 537 | VKILNAGRNPFRCTCELRDFIQLE |
| LRR-CT | 514 | 578 | VKILNAGRNPFRCTCELRDFIQLEEYSEGMMIGWLDSYICEYPLTLKGTLLKDVHLPELSCNTTL |
| Transmembrane | 579 | 601 | LIVTIVVIMLVLGMAVAFCCFYF |
| TIR | 602 | 807 | DLPWYLRMLGQWTLQRIRKTTQEQLKRNVQFHVFIAYSEHDSTWVKHELIPNLEKKEKLICLHEGNFDPGKSIIENIMNCIEKSYKSIFVLSPNFVQSEWCHYELYFAHHSLFHENSDYIIFILLEPIPLYCIPTKYPKLKALMEKKAYLEWPKDRRICGLFWANLRAAINANLLETREMYELQTFVGLNEESQGSAISLIRTDCL |
