# Supplementary material for: Characterization of the peripheral blood transcriptome and adaptive evolution of the MHC I and TLR gene families in the wolf (Canis lupus)
Source: BMC Genomics. 2017 Aug 7;18:584. doi: 10.1186/s12864-017-3983-0 (PMC5545864; doi:10.1186/s12864-017-3983-0)
Supplement: Supplementary file 9 — Species-specific positive selection sites in wolf TLR repertoire by MEME method. (DOCX 21 kb) [file 12864_2017_3983_MOESM9_ESM.docx]

Additional file 9: Table S17. Species-specific positive selection sites in wolf TLR repertoire by MEME method

| Gene | Site position | Sub-domain | Amino acid changes | Property changes | Functional information |
| --- | --- | --- | --- | --- | --- |
| TLR2 | 309 | LRR-10 | Gly-Gln | S, NP, NEU-P, NEU | adjacent to site involved in ligand binding [1] |
|  | 578 | LRR-CT | Asp-Thr | S, P, NEG-S, P, NEU |  |
|  | 609 | Transmembrane | Leu-Ala | NP, NEU- S, NP, NEU |  |
| TLR3 | 491 | LRR-18 | Val-Thr | NP, NEU-S, P, NEU | involved in to potential RNA binding [2] |
| TLR4 | 46 | LRR-NT | Asn-Ser | S, P, NEU -S, P, NEU |  |
|  | 306 | LRR-10 | Cys-Gln | S, NP, NEU-P, NEU |  |
| TLR5 | 428 | LRR-16 | Ile-Leu | NP, NEU-NP, NEU | involved in species-specific flagellin recognition [3] |
| TLR6 | 563 | LRR-CT | Tyr-Ser | P, NEU-S, P, NEU |  |
| TLR8 | 458 | LRR-14 | Ile-Met | NP, NEU-NP, NEU |  |
| TLR9 | 87 | LRR-1 | Ser-Val | S, P, NEU-NP, NEU |  |
|  | 504 | LRR-16 | Cys-Gly | S, NP, NEU-S, NP, NEU | near the potential ligand-binding residues [4] |

Notes: S, small; NP, nonpolar; NEU, neutral; P, polar; NEG, negatively charged.

References

1. Jin MS, Kim SE, Heo JY, Lee ME, Kim HM, Paik SG, Lee H, Lee JO: Crystal structure of the TLR1-TLR2 heterodimer induced by binding of a tri-acylated lipopeptide. *Cell* 2007, 130(6):1071-1082.

2. Choe J, Kelker MS, Wilson IA: Crystal structure of human toll-like receptor 3 (TLR3) ectodomain. *Science* 2005, 309(5734):581-585.

3. Andersen-Nissen E, Smith KD, Bonneau R, Strong RK, Aderem A: A conserved surface on Toll-like receptor 5 recognizes bacterial flagellin. *The Journal of experimental medicine* 2007, 204(2):393-403.

4. Wei T, Gong J, Jamitzky F, Heckl WM, Stark RW, Rossle SC: Homology modeling of human Toll-like receptors TLR7, 8, and 9 ligand-binding domains. Protein science : a publication of the Protein Society 2009, 18(8):1684-1691.
