# Supplementary material for: Characterization of the peripheral blood transcriptome and adaptive evolution of the MHC I and TLR gene families in the wolf (Canis lupus)
Source: BMC Genomics. 2017 Aug 7;18:584. doi: 10.1186/s12864-017-3983-0 (PMC5545864; doi:10.1186/s12864-017-3983-0)
Supplement: Supplementary file 10 — Wolf MHC I primer sets. (DOCX 19 kb) [file 12864_2017_3983_MOESM10_ESM.docx]

Additional file 10: Table S18. Wolf MHC I primer sets.

| Locus | Primer name | Primer sequence (5’→3’) | Size (bp) | Tm (°C) |
| --- | --- | --- | --- | --- |
| DLA-12 | DLA-12_F | CGGAACCCTAGCCCTGC | 1346 | 59 |
|  | DLA-12_R | GGCACTACACTCAGCCCAAC |  |  |
| DLA-64 | DLA-64_F | CGGAGATGGAGGTGGTGA | 654 | 57 |
|  | DLA-64_R | GGTGGCGGGTCAGGTAGATT |  |  |
| DLA-79 | DLA-79_F | GGCCCAGACCAGTGCA | 818 | 57 |
|  | DLA-79_R | TCAGGCTCTTGTGCAGAATAT |  |  |
| DLA-88 | DLA-88_F | CGGAGATGGAGGTGGTGA | 654 | 57 |
|  | DLA-88_R | GGTGGCGGGTCACACG |  |  |

The primers for DLA-88 were derived from *Ross* *et al*. [1] and the others were designed in this study.

References

1. Ross P, Buntzman AS, Vincent BG, Grover EN, Gojanovich GS, Collins EJ, Frelinger JA, Hess PR: Allelic diversity at the DLA-88 locus in Golden Retriever and Boxer breeds is limited. Tissue antigens 2012, 80(2):175-183.
